# Effect of preoperative oral isomaltulose on insulin resistance and glycemic variability in patients with type 2 diabetes mellitus: a randomized controlled trial

**DOI:** 10.3389/fnut.2025.1718843

**Published:** 2026-01-07

**Authors:** Chenyang Wang, Yue Shang, Jiameng Wang, Li Sun, Zhengmin Li, Changjun Gao

**Affiliations:** Department of Anesthesiology, Tangdu Hospital, The Fourth Military Medical University, Xi'an, Shaanxi, China

**Keywords:** glycemic variability, insulin resistance, isomaltulose, preoperative oral carbohydrate, type 2 diabetes mellitus

## Abstract

**Background/objectives:**

Preoperative oral carbohydrate alleviates postoperative insulin resistance and enhances recovery. Its utility in type 2 diabetes mellitus (T2DM) is debated due to hyperglycemia risks. Isomaltulose, a low-glycemic-index carbohydrate, has been shown to effectively attenuate postprandial glucose fluctuations and reduce metabolic stress on pancreatic β-cells in T2DM patients. This study investigated the effects of preoperative oral isomaltulose in patients with T2DM undergoing elective laparoscopic non-gastrointestinal surgery.

**Methods:**

60 T2DM patients were randomized to receive either 300 mL of a solution containing 50 g isomaltulose (isomaltulose group) or plain water (Control group) 2–3 h preoperatively. The primary outcome was insulin resistance, assessed by the homeostasis model assessment (HOMA-IR) at 24 h post surgery. Secondary outcomes included perioperative glycemic variability, serum insulin levels, Perlas grade (assessed by pre-anesthesia gastric ultrasound), incidence of regurgitation and aspiration, perioperative subjective comfort scores, postoperative nausea and vomiting incidence (PONV), time of first flatus after surgery, surgical wound complications, length of postoperative hospital stay.

**Results:**

Baseline characteristics were comparable. At 24 h postoperatively, the Isomaltulose group exhibited significantly lower HOMA-IR [6.89 ± 3.38 vs. 11.60 ± 4.34; mean difference = 4.71, 95% CI (2.70, 6.72), *p* < 0.001] and serum insulin levels [15.59 ± 5.58 vs. 24.54 ± 5.92 mU/L; mean difference = 8.94, 95% CI (5.97, 11.92), *p* < 0.001]. Although overall blood glucose was higher [155.70 (129.60–183.60) vs. 144.0 (113.40–182.25) mg/dL, *p* = 0.045], glycemic variability was significantly better [20.54% (16.19–26.69) vs. 27.95% (22.89–31.78), *p* < 0.001]. Gastric emptying (*p* = 0.24), patient comfort, PONV, and gastrointestinal recovery were similar. No regurgitation, aspiration, or serious complications occurred.

**Conclusion:**

In well-controlled T2DM patients, preoperative oral isomaltulose was associated with lower postoperative insulin resistance and reduced glycemic variability, without delaying gastric emptying. These metabolic benefits were accompanied by a transient elevation in pre-induction blood glucose, which should be weighed in clinical decision making.

**Clinical trial registration:**

https://www.chictr.org.cn/index.html, identifier ChiCTR2500098088.

## 1 Introduction

Preoperative oral carbohydrate (POC) is a key component of enhanced recovery after surgery (ERAS) protocols, known to alleviate postoperative insulin resistance (IR) (1, 2). However, its application in patients with type 2 diabetes mellitus (T2DM) remains controversial. Although preliminary evidence suggests it may be safe regarding gastric emptying (3), persistent concerns focus on glycemic fluctuations induced by commonly used maltodextrin-based formulations (4). Elevated glycemic variability in T2DM patients is associated with adverse outcomes (5, 6), which limits POC utilization in this population.

Isomaltulose, a natural disaccharide with a low glycemic index (GI = 32), offers a potential solution. Its unique α-1,6-glycosidic bond results in slow intestinal hydrolysis, leading to an attenuated postprandial glycemic and insulinemic response compared to rapidly absorbed carbohydrates (7, 8). Systematic reviews confirm its metabolic benefits in T2DM patients (9).

Given its potential to provide the metabolic benefits of POC while minimizing glycemic excursions, isomaltulose is a promising alternative for surgical patients with T2DM; however, its safety and efficacy in this setting remain unproven in randomized trials. This study therefore aims to investigate whether preoperative oral isomaltulose can improve postoperative insulin resistance while controlling glycemic variability in T2DM patients undergoing elective laparoscopic non-gastrointestinal surgery.

## 2 Materials and methods

### 2.1 Participants

This was an investigator-blinded randomized controlled trial approved by the Ethics Committee of Tangdu Hospital, The Fourth Military Medical University (Approval No. 202501-01) and registered at the Chinese Clinical Trial Registry (Registration No. ChiCTR2500098088) on March 3, 2025. All procedures involving human participants adhered to the ethical standards of the World Medical Association (Declaration of Helsinki). We prospectively enrolled patients with T2DM scheduled for elective laparoscopic non-gastrointestinal surgeries (including cholecystectomy, partial hepatectomy, hernia repair, and gynecological procedures) at our institution between March 10, 2025 and July 4, 2025, with written informed consent obtained from all participants. Elective laparoscopic non-gastrointestinal surgeries were selected to provide a controlled model of surgical metabolic stress while excluding the direct confounding effects of altered gastrointestinal anatomy and motility on the intervention. This model aligns with those commonly used in studies investigating preoperative oral carbohydrates.

The inclusion criteria were: (1) age 18–75 years; (2) diagnosed with T2DM; (3) random fasting blood glucose < 128 mg/dL; (4) glycated hemoglobin A1c (HbA1c) < 7%; (5) American Society of Anesthesiologists (ASA) physical status I-III; and (6) body mass index (BMI) 18–25 kg/m^2^. Patients were excluded if they met any of the following criteria: (1) history of upper gastrointestinal surgery; (2) high risk of regurgitation and aspiration (delayed/impaired gastric emptying, gastrointestinal obstruction, etc.); (3) severe metabolic disorders other than T2DM or other types of diabetes; (4) severe obesity or malnutrition; (5) communication or cognitive impairments preventing comprehension of the Visual Analogue Scale (VAS) scoring system.

Discontinuation Criteria included: (1) Participant-initiated withdrawal; (2) Modification of surgical schedule; (3) Intraoperative conversion to alternative surgical approaches; (4) Significant intraoperative hemorrhage (blood loss >1,000 mL); (5) Unplanned postoperative intensive care unit admission.

### 2.2 Grouping and intervention

The single study site (Tangdu Hospital, The Fourth Military Medical University) was selected based on its significant experience in elective laparoscopic surgeries, established enhanced recovery after surgery protocols, and possession of the necessary infrastructure and expertise to conduct the trial in strict accordance with the protocol. Upon hospital admission, potential participants were screened for eligibility by research staff. Written informed consent was obtained from all eligible individuals prior to enrollment completion. We established a panel comprising three patient advisors who reviewed and revised the trial protocol and informed consent documents to enhance their comprehensibility. Enrolled patients were randomly assigned in a 1:1 ratio using a computer-generated allocation sequence prepared by an independent statistician who was not involved in trial conduct. The allocation results were concealed in sequentially numbered, opaque, sealed envelopes. On the evening before surgery, a designated study coordinator opened the envelopes and assigned patients to either the isomaltulose or the control group accordingly. All participants were instructed to fast after 23:00 that night.

Patients allocated to the isomaltulose group received 300 mL of a solution containing 50 g of isomaltulose (Qingdao Honeycon Bio-Technology Co., Ltd., China) 2–3 h preoperatively, yielding a calculated concentration of 16.7% (w/v), while those in the control group consumed an equal volume of plain water following identical procedures. The 50 g dose of isomaltulose was selected as it is calorically equivalent to the carbohydrate load recommended in ERAS protocols and aligns with doses used in prior safety studies. The 2–3 h preoperative administration window follows standard ERAS guidelines to ensure adequate gastric emptying. The isomaltulose solution and plain water were administered by dedicated research nurses who were not involved in outcome assessment. These nurses were solely responsible for preparing and distributing the study solutions according to a standardized script, ensuring identical instructions for all participants. No additional oral intake was permitted on the day of surgery for either group. Due to the perceptible difference in taste between the interventions, participants and the nursing staff administering the solutions were not blinded. To minimize performance bias, the nurses’ role was strictly limited to intervention administration; they did not participate in any data collection, outcome evaluation, or analysis. However, outcome assessors (including those collecting subjective scores and postoperative data, as well as the anesthesiologists performing gastric ultrasonography), care providers involved in postoperative management, and data analysts remained blinded to group allocation throughout the trial. The allocation sequence was concealed from all blinded personnel until the completion of data analysis.

### 2.3 Perioperative management and anesthesia protocol

Upon arrival in the operating room, all patients underwent standard monitoring including electrocardiogram (ECG), pulse oximetry (SpO₂), invasive arterial blood pressure via radial artery cannulation, core temperature, and bispectral index (BIS). After preoxygenation, general anesthesia was induced with intravenous midazolam (0.05 mg/kg), etomidate (0.3 mg/kg), sufentanil (0.3–0.5 μg/kg), and rocuronium (0.6 mg/kg). Tracheal intubation was performed using video laryngoscopy, followed by mechanical ventilation in pressure-controlled volume-guaranteed (PCV-VG) mode with tidal volumes of 6–8 mL/kg, adjusted to maintain the end-tidal carbon dioxide partial pressure (PetCO₂) at 35–45 mmHg. Anesthesia maintenance consisted of sevoflurane (1.5–2% end-tidal concentration) with continuous infusions of remifentanil (0.2–0.5 μg/kg/min) and dexmedetomidine (0.2–0.7 μg/kg/h), supplemented by intermittent rocuronium (15–20 mg) under train-of-four monitoring. Multimodal warming strategies maintained normothermia throughout the procedure. Post-induction ultrasound-guided bilateral transversus abdominis plane (TAP) blocks were performed using 20 mL of 0.25% ropivacaine per side.

Upon surgery completion, patients were transferred to the post-anesthesia care unit (PACU) and extubated after achieving predefined criteria: full consciousness, stable spontaneous ventilation (tidal volume >6 mL/kg), hemodynamic stability, and train-of-four ratio ≥0.9. PACU discharge required an Aldrete score ≥9. Postoperative analgesia was provided via patient-controlled intravenous analgesia (PCIA) containing sufentanil 100 μg and tropisetron 5 mg in 100 mL normal saline, programmed with a 2.5 mL/h basal infusion, 2.0 mL bolus dose, and 15-min lockout interval. Patients were encouraged to ambulate early postoperatively.

### 2.4 Data collection

The baseline data included demographic characteristics: age, sex, BMI, ASA classification, duration of diabetes, preoperative albumin levels, preoperative hemoglobin levels, HbA1c, and random fasting blood glucose levels, fasting duration. Intraoperative data encompassed surgical procedure type, operative time, intraoperative blood loss, urine output, and intraoperative fluid administration volume.

The primary outcome was postoperative insulin resistance. Secondary outcomes included perioperative glycemic variability, serum insulin levels, Perlas grade (assessed by pre-anesthesia gastric ultrasound), incidence of regurgitation and aspiration (from anesthesia induction to first 24 postoperative hours), perioperative subjective comfort scores, postoperative nausea and vomiting incidence (PONV), time of first flatus after surgery, surgical wound complications, length of postoperative hospital stay.

Capillary blood glucose levels were measured at the following time points: before oral liquid on the day of surgery (T1), before anesthesia induction (T2), surgical completion (T3), 6 h postoperatively (T4), and 24 h postoperatively (T5). Capillary blood glucose was measured using an ACCU-CHEK Performa glucometer (Roche Diagnostics, Germany). Quality control testing was performed in accordance with the manufacturer’s instructions at the study outset and with each new vial of test strips. All measurements were taken by a single trained research nurse using a standardized technique to ensure consistency. Serum insulin levels were measured at T1 and T5 time points. Insulin resistance was evaluated using the homeostasis model assessment (HOMA-IR), calculated at T1 and T5 time points with the following formula: HOMA-IR = [blood glucose (mmol/L) × serum insulin (mU/L)]/22.5. Glycemic variability was quantified using the coefficient of variation (CV), calculated as: (standard deviation of blood glucose/mean blood glucose) × 100%. CV was selected as the most appropriate metric for assessing glycemic variability in this context for two reasons. First, compared to the standard deviation (SD), which is an absolute measure of spread, CV is a scale-independent relative measure. This property is essential for validly comparing glycemic variability between our two study groups, which exhibited different overall glycemic levels. Second, the mean amplitude of glycemic excursions (MAGE), while established, is a metric optimized for and typically calculated from continuous glucose monitoring (CGM) data. Since our protocol employed intermittent capillary blood sampling rather than CGM, MAGE was not a feasible option. The Perlas grade system, developed by Perlas et al. (10), is a three-tier classification method based on qualitative assessment of the gastric antrum that reliably predicts perioperative aspiration risk. Prior to anesthesia induction, gastric ultrasound examinations were performed by a single attending anesthesiologist who was specifically trained in perioperative gastric sonography and blinded to group allocation. For each patient, a minimum of three consecutive images of the gastric antrum were obtained in both the supine and right lateral decubitus positions. The grading criteria were as follows: Grade 0 indicated no fluid present in either position; Grade 1 indicated fluid visible only in the right lateral position; and Grade 2 indicated fluid present in both positions, with higher grades corresponding to greater aspiration risk. All acquired ultrasound images were printed and archived within the patient’s individual Case Report Form (CRF) for verification.

Thirst and hunger were assessed using a 10 cm VAS, where 0 indicated no discomfort and 10 indicated the most severe discomfort. A blinded anesthesiologist evaluated these parameters before anesthesia induction and before discharge from the PACU.

Any unfavorable and unintended sign, symptom, or disease occurring during the trial period was defined as an adverse event. We specifically defined hyperglycemia as a blood glucose level >250 mg/dL (requiring immediate insulin intervention) and hypoglycemia as a blood glucose level <70 mg/dL. Additionally, a systematic and multi-phase monitoring protocol for regurgitation and aspiration was rigorously implemented throughout the perioperative period. During anesthesia induction, continuous oropharyngeal inspection and post-intubation laryngeal visualization using video laryngoscopy were performed to detect any gastric content leakage. Throughout emergence, patients were closely monitored for clinical signs of aspiration including coughing, laryngospasm, oxygen desaturation >5%, or new respiratory auscultation findings. Postoperatively, all patients were followed for 24 h with daily chart reviews and structured interviews to identify any delayed symptoms suggestive of aspiration pneumonia, with provision for immediate radiographic confirmation if indicated. This protocol was designed to facilitate active detection and reporting of these endpoints. However, the ability to draw definitive conclusions about the incidence of such rare events from a single trial of this size is inherently limited.

### 2.5 Sample size

The sample size calculation was based on detecting a 2.0-point difference in HOMA-IR between groups, an effect size derived from a previous randomized trial in T2DM patients undergoing laparoscopic cholecystectomy (11). Using PASS 15.0 software (Stata Corp. LP, College Station, TX) with a 1:1 allocation ratio, *α* = 0.05, and 90% power, the calculated sample size was 25 patients per group (total *n* = 50). To minimize potential errors, enhance statistical power, and account for possible dropouts, we ultimately enrolled 60 patients (30 per group).

### 2.6 Data analysis

Statistical analyses were performed using SPSS 26.0 (IBM Corp). The normality of distribution for all continuous variables was assessed using the Shapiro–Wilk test. Based on this assessment, data are presented as mean ± SD for normally distributed variables or median (interquartile range [IQR]) for non-normally distributed variables, with group comparisons made using independent t-tests or Mann–Whitney U tests, respectively. Categorical variables were compared using *χ*^2^ or Fisher’s exact tests. For the longitudinal blood glucose data, the overall group-by-time interaction was analyzed using generalized estimating equations (GEE) with an exchangeable (compound symmetry) working correlation structure. This structure is parsimonious and represents the conventional, recommended choice for analyzing repeated measures over a short period with a limited number of fixed time points. To explore the source of significant overall effects, *a priori* pairwise comparisons between groups at individual time points were conducted, with *p*-values adjusted using the Benjamini-Hochberg (BH) correction for multiple comparisons. Statistical significance was set at *p* < 0.05.

## 3 Results

### 3.1 Baseline characteristics

**Figure 1:**
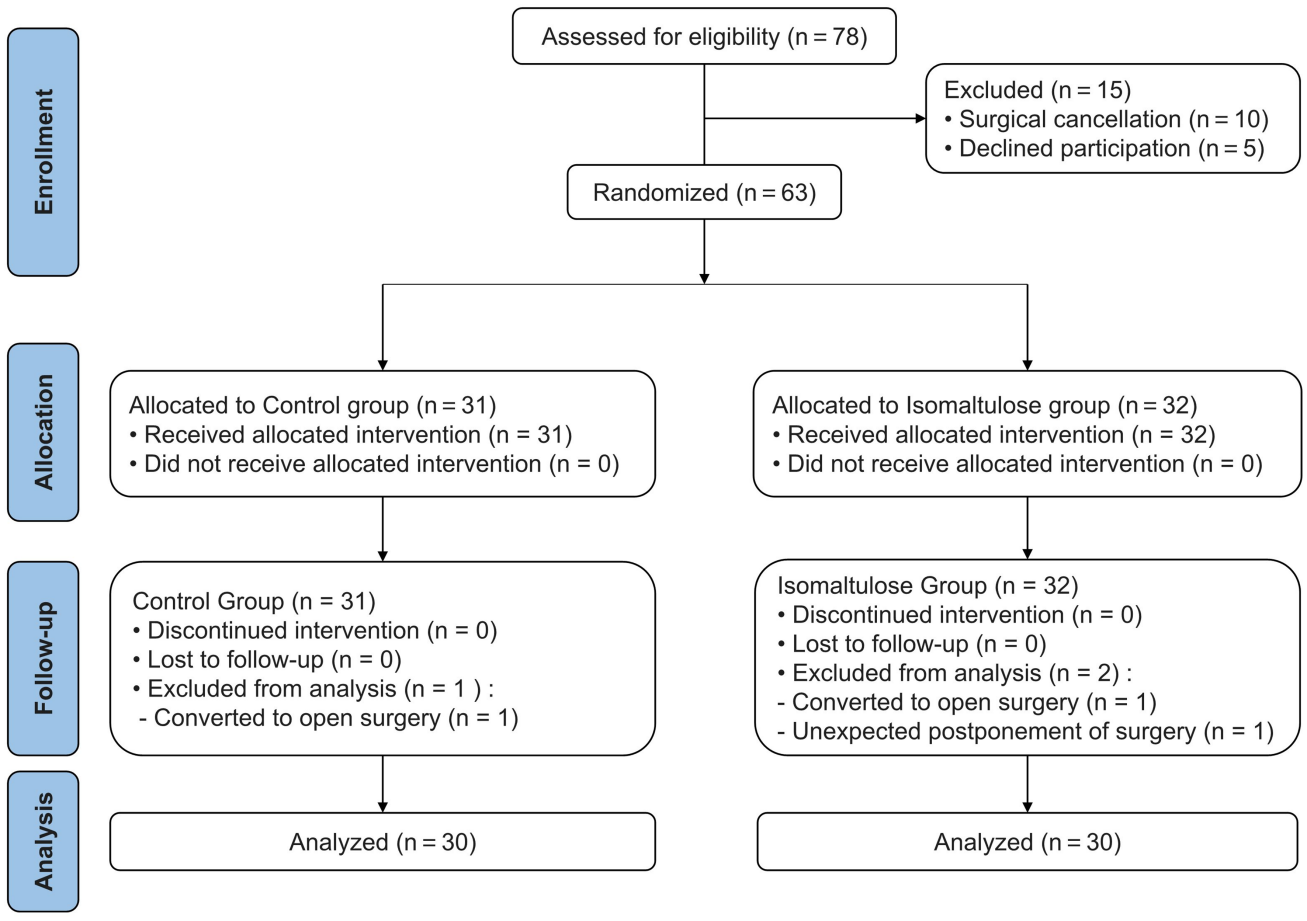
The consolidated standards of reporting trials (CONSORT) flow diagram.

During the study period, 78 patients were initially assessed for eligibility. Among these, 10 patients were excluded due to surgical cancellation and 5 declined participation, resulting in 63 patients being enrolled and randomly allocated to either the control group (*n* = 31) or the isomaltulose group (*n* = 32). Subsequently, one patient in the control group was excluded due to conversion to open surgery, while two patients in the isomaltulose group were excluded: one due to conversion to open surgery and one due to unexpected postponement of surgery. Consequently, 30 patients in each group completed the study and were included in the final analysis (Figure 1). There were no missing data for the primary or secondary outcomes. All randomized participants (*n* = 60) completed the study protocol and provided complete data for all prescribed assessments. All analyzed patients demonstrated comparable baseline characteristics and intraoperative parameters (Table 1).

**Table 1:** Baseline and intraoperative characteristics.

Characteristic	Control group (*n* = 30)	Isomaltulose group (*n* = 30)	*p*-Value
Age (years)	63.0 (56.0, 69.0)	60.5 (55.6, 68.3)	0.58
Male/female	9/21	10/20	0.78
BMI (kg/m^2^)	22.0 (21.2, 23.0)	22.8 (21.5, 24.3)	0.12
ASA classification			0.74
II	25 (83.3%)	24 (80.0%)	
III	5 (16.7%)	6 (20.0%)	
Diabetes duration (years)	2.0 (1.0, 5.0)	3.0 (1.8, 10.0)	0.36
Albumin (g/dL)	4.4 ± 0.2	4.3 ± 0.2	0.14
Hemoglobin (g/dL)	14.4 ± 1.2	14.0 ± 1.0	0.19
Random fasting glucose (mg/dL)	103.6 ± 10.3	107.8 ± 10.2	0.11
HbA1c (%)	5.9 (5.3, 6.5)	6.3 (5.6, 6.8)	0.16
Fasting duration (min)	975.0 (915.0, 1170.0)	1089.0 (892.5, 1159.5)	0.59
Fluid fasting duration (min)	146.6 ± 7.8	149.9 ± 8.3	0.12
Surgical procedures			0.97
Cholecystectomy	14 (46.6%)	16 (53.3%)	
Hysterectomy with bilateral salpingo-oophorectomy	6 (20.0%)	5 (16.7%)	
Herniorrhaphy	3 (10.0%)	3 (10.0%)	
Partial hepatectomy	3 (10.0%)	2 (6.7%)	
Salpingo-oophorectomy	2 (6.7%)	3 (10.0%)	
Hysterectomy	2 (6.7%)	1 (3.3%)	
Operative time (min)	92.0 (64.3, 155.5)	92.5 (68.8, 162.5)	0.65
Intraoperative blood loss (mL)	20.0 (10.0, 50.0)	20.0 (10.6, 62.5)	0.79
Intraoperative urine output (mL)	150.0 (0, 325.0)	200.0 (137.5, 325.0)	0.29
Intraoperative fluid administration (mL)	1000.0 (600.0, 1500.0)	1000.0 (775.0, 1500.0)	0.71

Data are presented as mean ± standard deviation (SD) or median [interquartile range (IQR)].

### 3.2 Insulin resistance

**Figure 2:**
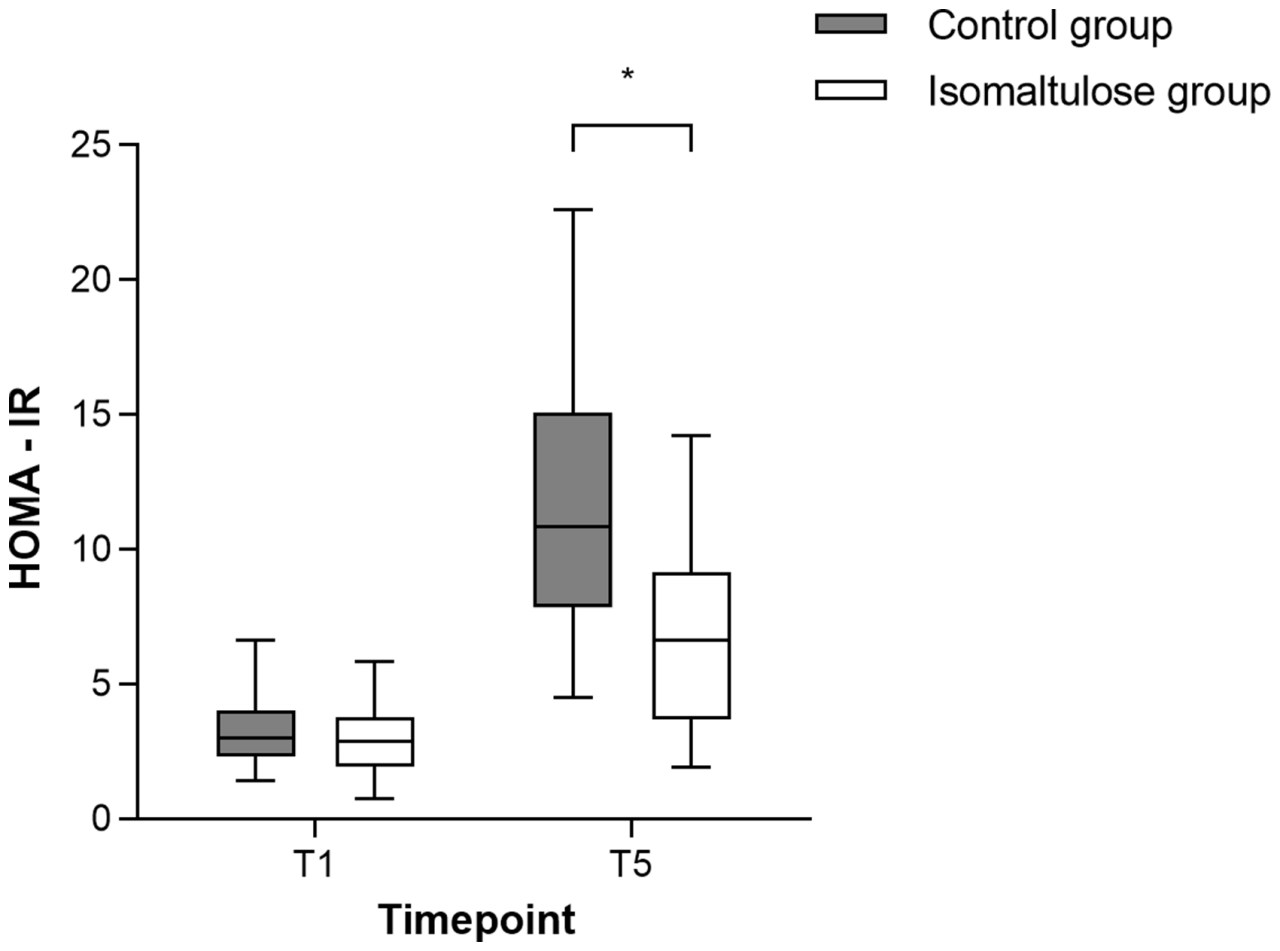
Comparison of HOMA-IR values before solution intake (T1) and at 24 h postoperatively (T5) in study subjects. *p < 0.001 vs. control at T5 (mean difference = 4.71, 95% CI 2.70–6.72). Each data point represents an individual patient.

At T1, no significant difference in HOMA-IR was found between the isomaltulose and control groups (2.95 ± 1.32 vs. 3.30 ± 1.31, *p* = 0.31). Although postoperative HOMA-IR increased significantly in both groups, the isomaltulose group maintained significantly lower values than the control group (Figure 2), with this difference being statistically significant [6.89 ± 3.38 vs. 11.60 ± 4.34; mean difference = 4.71, 95% CI (2.70, 6.72), *p* < 0.001].

### 3.3 Insulin levels

**Figure 3:**
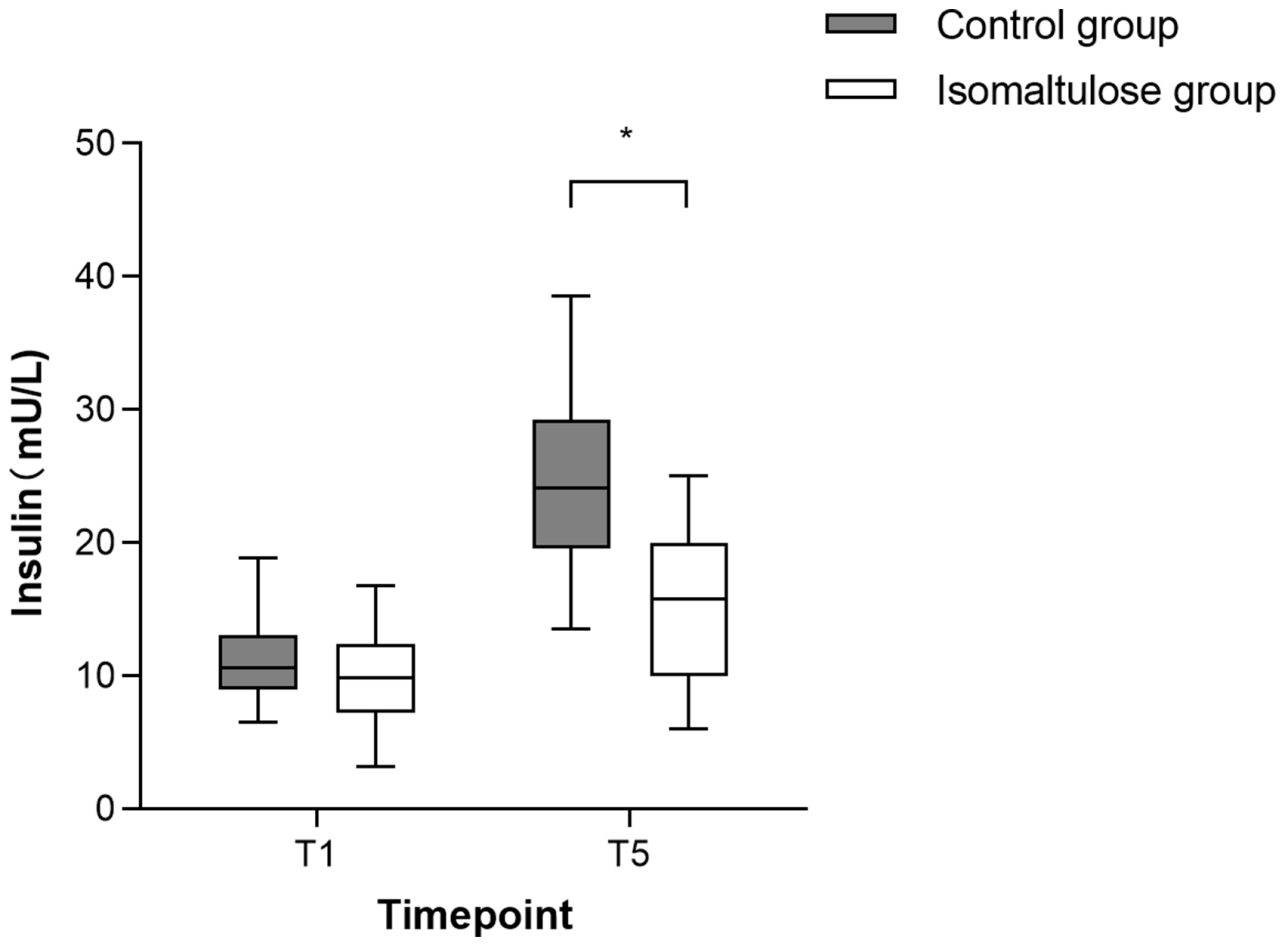
Serum insulin levels before solution intake (T1) and 24 h postoperatively (T5) in study subjects. *p < 0.001 vs. control at T5 (mean difference = 8.94, 95% CI 5.97–11.92). Each data point represents an individual patient.

Insulin levels did not differ between groups at T1 (9.87 ± 3.41 vs. 11.38 ± 3.08 mU/L, *p* = 0.78). Postoperatively, while both groups showed elevated levels (Figure 3), the isomaltulose group demonstrated significantly lower insulin than the control group [15.59 ± 5.58 vs. 24.54 ± 5.92 mU/L; mean difference = 8.94, 95% CI (5.97, 11.92), *p* < 0.001].

### 3.4 Blood glucose fluctuations

**Figure 4:**
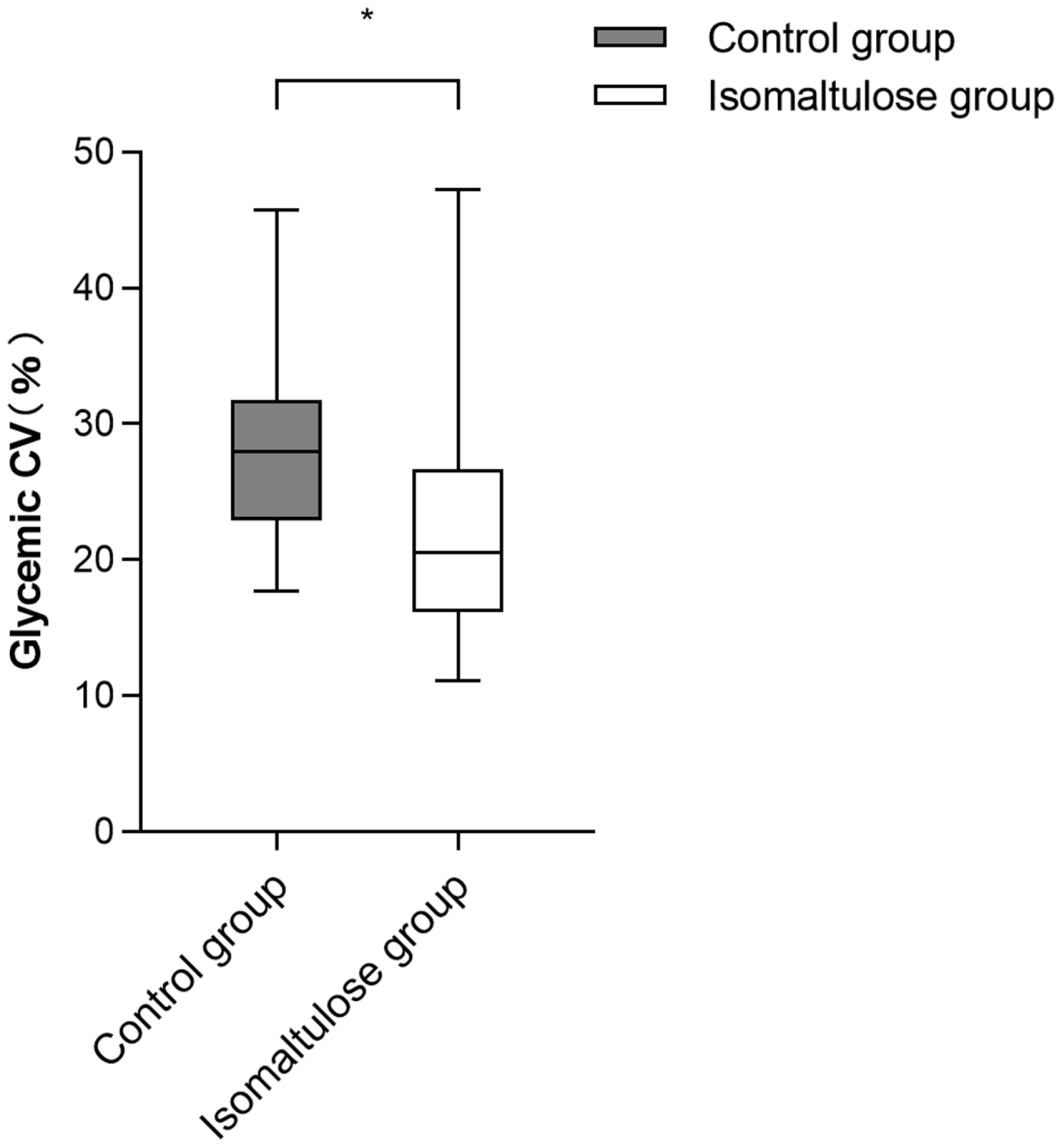
Perioperative glycemic variability in study subjects. Glycemic variability is expressed as the coefficient of variation (CV). The isomaltulose group showed a significantly lower CV (*p < 0.001, Mann–Whitney U test). Each data point represents an individual patient.

The isomaltulose group demonstrated earlier-onset but more gradual blood glucose elevation. CV was significantly lower in the isomaltulose group compared to the control group [median (IQR), 20.54% (16.19–26.69) vs. 27.95% (22.89–31.78), *p* < 0.001; Figure 4].

**Figure 5:**
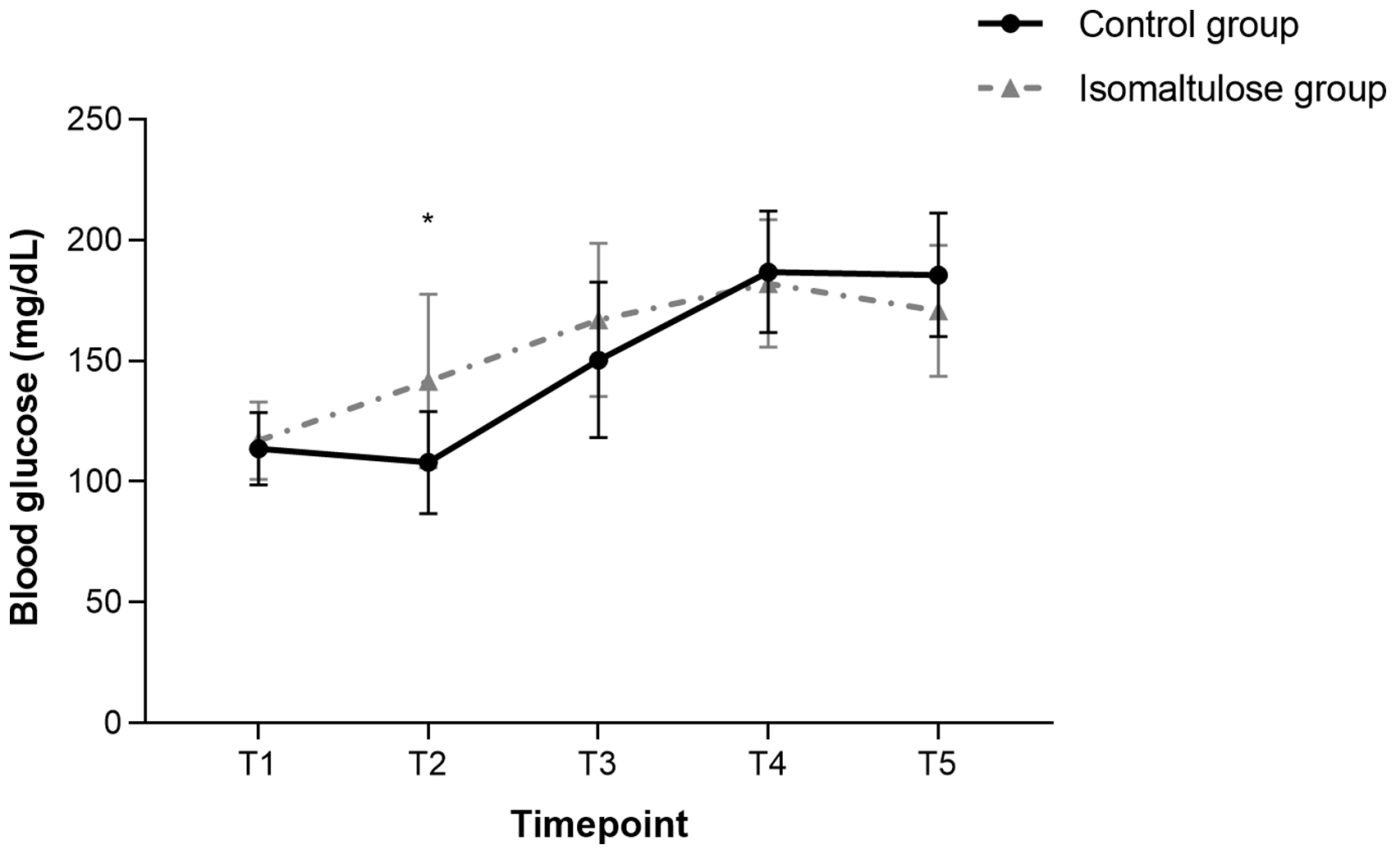
Perioperative blood glucose levels. Time points: T1, pre-intervention (surgery day); T2, pre-induction; T3, surgical completion; T4, 6 h postoperative; T5, 24 h postoperative. *BH-adjusted *p* < 0.001 vs. control at T2 (mean difference = 33.72 mg/dL, 95% CI 18.40–49.04). Each data point represents an individual patient.

It is clinically noteworthy that, despite lower glycemic variability, the isomaltulose group had higher overall blood glucose levels [median (IQR), 155.70 (129.60–183.60) vs. 144.0 (113.40–182.25) mg/dL; GEE, *p* = 0.045], with a significant elevation observed specifically at T2 [141.67 ± 35.53 vs. 107.94 ± 21.20 mg/dL; mean difference = 33.72 mg/dL, 95% CI (18.40, 49.04), BH-adjusted *p* < 0.001; Figure 5]. At this time point, 4 out of 30 patients (13.3%) in the isomaltulose group had blood glucose levels ≥180 mg/dL, with a maximum of 201.6 mg/dL, whereas all values in the control group were below this threshold (Supplementary Table S1).

### 3.5 Other outcome measures

The isomaltulose group demonstrated slightly higher pre-induction Perlas grade than the control group, and the difference was not statistically significant. No episodes of regurgitation or aspiration were detected under the study’s monitoring protocol. Subjective assessment scores were similar between groups for hunger, while thirst scores showed mild postoperative increases from baseline in both groups without significant intergroup differences. Although postoperative nausea incidence was somewhat higher in controls compared to the isomaltulose group, this difference was not statistically significant, nor were there differences in vomiting incidence or postoperative hospital stay. Similarly, the two groups showed no significant difference in the time of first flatus after surgery (Table 2). During follow-up, only one case of surgical site infection occurred (in the isomaltulose group), with no other adverse outcomes observed in either group.

**Table 2:** Other secondary outcomes in study participants.

Outcome	Control group (*n* = 30)	Isomaltulose group (*n* = 30)	*p*-Value
Perlas grade			0.24
Grade 0	23 (76.7%)	19 (63.4%)	
Grade 1	7 (23.3%)	10 (33.3%)	
Grade 2	0 (0%)	1 (3.3%)	
Incidence of regurgitation and aspiration	0 (0%)	0 (0%)	
Hunger (VAS)			
Pre-induction	2.0 (1.0, 2.0)	1.5 (0, 2.0)	0.47
Postoperative	1.0 (0, 2.0)	1.0 (0, 1.0)	0.61
Thirst (VAS)			
Pre-induction	2.0 (1.0, 2.0)	2.0 (1.0, 2.0)	0.73
Postoperative	3.0 (2.0, 4.0)	3.0 (2.0, 4.0)	0.72
Nausea	5 (16.7%)	2 (6.7%)	0.23
Vomiting	0 (0%)	0 (0%)	
Time of first flatus after surgery (hours)	20.0 (18.0, 25.3)	18.0 (16.0, 23.3)	0.14
Wound complications	0 (0%)	1 (3.3%)	0.31
Postoperative hospital stay (days)	3.0 (2.0, 6.0)	2.0 (2.0, 6.0)	0.90

Data are presented as mean ± SD or median (IQR).

## 4 Discussion

Our results demonstrated that preoperative oral isomaltulose significantly reduced postoperative insulin resistance in T2DM patients. Moreover, it effectively mitigated perioperative glycemic fluctuations without delaying gastric emptying. These findings provide a novel nutritional intervention strategy for perioperative management in T2DM patients.

The attenuation of postoperative insulin resistance observed in our study aligns with the established benefits of preoperative oral carbohydrate (12–14), an effect that may be related to POC’s ability to improve catabolic status and mitigate surgical stress (15). Our findings extend this evidence by demonstrating that isomaltulose can achieve this benefit in well-controlled T2DM patients undergoing laparoscopic surgery. Specifically, the reduction in HOMA-IR suggests a mitigation of postoperative insulin resistance with potential clinical benefits, including faster recovery of metabolic homeostasis, reduced protein catabolism, and potentially shorter convalescence (16). Notably, isomaltulose may further optimize these effects through the unique incretin modulation (17, 18), which differs from that of rapidly absorbed carbohydrates. Furthermore, the concentration of the isomaltulose solution used in our study (16.7% w/v) is comparable to concentrations (e.g., 18%) that have been shown effective in alleviating preoperative insulin resistance in other studies, supporting the physiological plausibility of our findings (19).

Current evidence suggests that POC may elevate blood glucose levels in diabetic patients. In a randomized controlled trial (20), approximately one-third of 49 T2DM patients receiving preoperative carbohydrate loading required insulin intervention, with a perioperative hyperglycemia incidence of 12.2%. Although Li et al. (21) attempted to optimize glycemic control through individualized insulin supplementation, the mean blood glucose levels in T2DM patients receiving carbohydrate loading remained higher than those in the strict fasting group. Additionally, carbohydrate intake may increase perioperative glycemic variability. This is supported by a prospective study in T2DM patients (22), which demonstrated that T2DM patients consuming carbohydrates 2–3 h preoperatively exhibited a 6.4% greater glucose variability compared to the control group.

In contrast, isomaltulose significantly reduces postprandial glucose peaks (approximately 25% lower than rapidly absorbed carbohydrates) due to its slow absorption properties (23). The results of this study demonstrated that although the overall blood glucose levels in the isomaltulose group were higher than those in the control group, all values remained within the controllable range without requiring insulin intervention. Importantly, the isomaltulose group showed more gradual perioperative glucose fluctuations, resulting in a significant reduction in CV compared to the control group. This reduction resulted in a median CV value that lies further from the established threshold for unstable glycemic control (CV > 36%) than the median value in the control group, indicating a shift towards greater glycemic stability (24).

However, a balanced clinical interpretation of our findings must acknowledge the concomitant elevation in pre-induction blood glucose (T2) observed in the isomaltulose group. This elevation was not merely statistical; clinically, 13.3% of patients in this group had values reaching or exceeding 180 mg/dL, a level that meets the American Diabetes Association’s common threshold for perioperative hyperglycemia requiring attention. This phenomenon was not observed in the control group. These data illustrate a distinct metabolic trade-off: preoperative isomaltulose provided benefits in terms of attenuated postoperative insulin resistance and improved glycemic stability, but at the cost of a predictable pre-induction glucose rise that was clinically significant in a subset of patients. Importantly, within the context of our study protocol involving well-controlled T2DM patients, these elevations were transient and did not necessitate unplanned insulin intervention. This suggests that for similar patients, the intervention may be manageable. Nonetheless, it unequivocally underscores the necessity for vigilant preoperative glucose monitoring if isomaltulose is used. The clinical decision must therefore weigh the demonstrated metabolic advantages against the need to monitor for and be prepared to manage this expected pre-procedural effect.

Existing research has demonstrated that patients with advanced-stage diabetes or acute hyperglycemia may exhibit delayed gastric emptying due to impaired autonomic nervous function (25). However, in well-controlled T2DM patients, gastric emptying function typically remains unaffected and may even surpass that of healthy individuals in the same age group (26, 27). To evaluate the safety of preoperative carbohydrate beverage intake in T2DM patients, Lee et al. (20) employed ultrasound examination to confirm that consumption of 200 mL of a 12.8% maltodextrin-containing complex carbohydrate beverage 2 h preoperatively neither increased gastric volume nor elevated the risk of regurgitation and aspiration during general anesthesia. This finding is supported by a systematic review (3), which concluded that intake of 200–400 mL of 12.5% carbohydrate solution 2–3 h preoperatively is safe for well-controlled T2DM patients.

Although existing studies have employed varying types, concentrations, and dosages of carbohydrates, current evidence preliminarily supports the feasibility of POC in T2DM patients. Furthermore, a sports medicine study (28) reported comparable gastric emptying rates between isomaltulose and maltodextrin during exercise. Based on this evidence, our study standardized the carbohydrate intake window to 2–3 h preoperatively. Our results are consistent with this literature, as no cases of regurgitation or aspiration were observed in this cohort of well-controlled T2DM patients. This adds to the growing body of evidence suggesting that preoperative oral isomaltulose, administered under a standardized protocol, may be metabolically feasible and generally well-tolerated in this population. Nevertheless, the statistical power of this study is insufficient to draw definitive conclusions about the risk of rare adverse events like aspiration, a point elaborated in the limitations part of the Discussion.

In this study, the control group received plain water as a reference. Due to the limited satiety effect of carbohydrates (29), the isomaltulose group did not demonstrate significant improvements in subjective sensations such as thirst and hunger. Furthermore, although previous studies (21) reported that preoperative carbohydrate supplementation could reduce the incidence of PONV in T2DM patients, our study failed to observe similar effects. This discrepancy may be attributed to our postoperative analgesia protocol, which included tropisetron, a medication with known antiemetic properties that might have masked the potential impact of carbohydrate intervention on PONV.

Previous studies have indicated that preoperative carbohydrate intake may facilitate postoperative recovery and reduce hospitalization duration (30), but this effect was not observed in our investigation. Time of first flatus after surgery is commonly used as an indicator of postoperative gastrointestinal recovery (31); however, our study also found no significant difference between the two groups. This discrepancy may be attributed to both the surgical types included in our study and the limited sample size. All procedures selected for this trial were laparoscopic surgeries, which inherently involve less trauma compared to open surgeries. Furthermore, the restricted sample size in our study might have prevented the detection of significant intergroup differences.

This study has several limitations. First, participants were not blinded to group allocation due to the perceptible taste of isomaltulose, which may have introduced bias into subjective measures. Second, the trial was powered specifically for its primary outcome, and consequently lacked sufficient power to detect significant differences in secondary outcomes or in the incidence of rare adverse events such as aspiration. While no such events were observed under our monitoring protocol, this finding should be interpreted as preliminary safety data within the constraints of the study’s sample size and not as conclusive evidence of safety. Third, this was a single-center study with a limited diversity of surgical procedures (elective laparoscopic non-gastrointestinal surgeries), and thus our findings are most directly applicable to similar minimally invasive procedures. Nevertheless, the underlying principle of modulating preoperative carbohydrate quality may have broader relevance to other surgical categories, though this requires validation in more diverse populations. Fourth, the follow-up period was short (24 h), limiting our assessment of longer-term metabolic outcomes. Fifth, glycemic monitoring was restricted to five predetermined time points. While this captured key perioperative phases, the absence of CGM means that transient glucose fluctuations may have been missed, limiting the ability of the CV to fully characterize glycemic excursions. Sixth, the study employed a fixed-dose, single-carbohydrate supplementation protocol without comparisons of different dosages or timing of administration. Seventh, the inclusion criteria were restricted to normal-weight (BMI 18–25 kg/m^2^), well-controlled (HbA1c < 7%) patients with T2DM, which limits the generalizability of our findings. Consequently, the applicability of our results to individuals with suboptimal glycemic control, insulin-dependent diabetes, or obesity remains unknown and warrants future investigation. Eighth, the study population was relatively homogeneous in terms of glycemic control and body weight; therefore, exploratory subgroup analyses by factors such as sex or age were not performed.

Future research should focus on the following: First, expanding population validation by assessing its safety and metabolic benefits in overweight/obese individuals, those with suboptimal glycemic control, and patients with insulin-dependent diabetes. Second, optimizing monitoring methods through the use of CGM to accurately quantify its impact on glycemic variability. Third, exploring protocol optimization via large-scale, multicenter trials to compare different dosages and timing regimens, and to investigate its synergistic effects when combined with other ERAS measures. These investigations will provide high-level evidence to inform individualized perioperative carbohydrate management strategies.

## 5 Conclusion

In well-controlled T2DM patients (specifically those with HbA1c < 7% and of normal weight), preoperative oral isomaltulose was associated with lower postoperative insulin resistance and reduced perioperative glycemic variability. The clinical application of these metabolic benefits should be considered alongside the concurrently observed elevation in pre-induction blood glucose.

## Data Availability

The raw data supporting the conclusions of this article will be made available by the authors, without undue reservation.
