# Supplementary material for: Effect of preoperative oral isomaltulose on insulin resistance and glycemic variability in patients with type 2 diabetes mellitus: a randomized controlled trial
Source: Front Nutr. 2026 Jan 7;12:1718843. doi: 10.3389/fnut.2025.1718843 (PMC12819711; doi:10.3389/fnut.2025.1718843)
Supplement: Supplementary file 1 [file Table_1.docx]

**Supplementary Table S1.** Individual capillary blood glucose readings (mg/dL) for all 60 randomized participants at five perioperative time points (T1: pre-intervention; T2: pre-induction; T3: surgical completion; T4: 6h postoperative; T5: 24h postoperative).

| Patient ID | Group | T1 (mg/dL) | T2 (mg/dL) | T3 (mg/dL) | T4 (mg/dL) | T5 (mg/dL) |
| --- | --- | --- | --- | --- | --- | --- |
| C-1 | Control | 120.6 | 118.8 | 95.4 | 156.6 | 190.8 |
| C-2 | Control | 93.6 | 73.8 | 109.8 | 189.0 | 216.0 |
| C-3 | Control | 133.2 | 93.6 | 124.2 | 212.4 | 183.6 |
| C-4 | Control | 104.4 | 91.8 | 145.8 | 183.6 | 135.0 |
| C-5 | Control | 113.4 | 124.2 | 174.6 | 185.4 | 162.0 |
| C-6 | Control | 88.2 | 81.0 | 138.6 | 167.4 | 160.2 |
| C-7 | Control | 127.8 | 120.6 | 149.4 | 201.6 | 214.2 |
| C-8 | Control | 111.6 | 95.4 | 160.2 | 171.0 | 162.0 |
| C-9 | Control | 100.8 | 104.4 | 127.8 | 201.6 | 163.8 |
| C-10 | Control | 142.2 | 131.4 | 167.4 | 167.4 | 214.2 |
| C-11 | Control | 124.2 | 138.6 | 120.6 | 208.8 | 237.6 |
| C-12 | Control | 95.4 | 82.8 | 131.4 | 205.2 | 172.8 |
| C-13 | Control | 120.6 | 127.8 | 225.0 | 210.6 | 169.2 |
| C-14 | Control | 127.8 | 113.4 | 174.6 | 153.0 | 167.4 |
| C-15 | Control | 108.0 | 88.2 | 153.0 | 192.6 | 162.0 |
| C-16 | Control | 113.4 | 129.6 | 189.0 | 212.4 | 221.4 |
| C-17 | Control | 129.6 | 111.6 | 178.2 | 160.2 | 196.2 |
| C-18 | Control | 108.0 | 102.6 | 106.2 | 194.4 | 217.8 |
| C-19 | Control | 99.0 | 86.4 | 117.0 | 181.8 | 217.8 |
| C-20 | Control | 122.4 | 97.2 | 160.2 | 198.0 | 212.4 |
| C-21 | Control | 131.4 | 135.0 | 156.6 | 207.0 | 212.4 |
| C-22 | Control | 117.0 | 136.8 | 203.4 | 163.8 | 189.0 |
| C-23 | Control | 102.6 | 117.0 | 171.0 | 219.6 | 201.6 |
| C-24 | Control | 115.2 | 99.0 | 135.0 | 205.2 | 176.4 |
| C-25 | Control | 136.8 | 140.4 | 192.6 | 226.8 | 185.4 |
| C-26 | Control | 86.4 | 77.4 | 102.6 | 167.4 | 162.0 |
| C-27 | Control | 126.0 | 84.6 | 113.4 | 174.6 | 160.2 |
| C-28 | Control | 91.8 | 79.2 | 142.2 | 111.6 | 162.0 |
| C-29 | Control | 115.2 | 133.2 | 181.8 | 167.4 | 163.8 |
| C-30 | Control | 104.4 | 122.4 | 163.8 | 210.6 | 180.0 |
| I-1 | Isomaltulose | 91.8 | 91.8 | 135.0 | 192.6 | 153.0 |
| I-2 | Isomaltulose | 113.4 | 154.8 | 196.2 | 180.0 | 230.4 |
| I-3 | Isomaltulose | 109.8 | 135.0 | 189.0 | 223.2 | 196.2 |
| I-4 | Isomaltulose | 100.8 | 99.0 | 149.4 | 183.6 | 212.4 |
| I-5 | Isomaltulose | 140.4 | 183.6 | 217.8 | 198.0 | 167.4 |
| I-6 | Isomaltulose | 124.2 | 129.6 | 163.8 | 207.0 | 149.4 |
| I-7 | Isomaltulose | 117.0 | 147.6 | 203.4 | 147.6 | 144.0 |
| I-8 | Isomaltulose | 129.6 | 162.0 | 192.6 | 163.8 | 181.8 |
| I-9 | Isomaltulose | 106.2 | 117.0 | 129.6 | 160.2 | 149.4 |
| I-10 | Isomaltulose | 120.6 | 124.2 | 153.0 | 140.4 | 153.0 |
| I-11 | Isomaltulose | 136.8 | 201.6 | 194.4 | 230.4 | 163.8 |
| I-12 | Isomaltulose | 95.4 | 138.6 | 144.0 | 208.8 | 160.2 |
| I-13 | Isomaltulose | 126.0 | 158.4 | 171.0 | 219.6 | 192.6 |
| I-14 | Isomaltulose | 104.4 | 86.4 | 106.2 | 158.4 | 185.4 |
| I-15 | Isomaltulose | 133.2 | 180.0 | 162.0 | 151.2 | 133.2 |
| I-16 | Isomaltulose | 108.0 | 104.4 | 120.6 | 178.2 | 138.6 |
| I-17 | Isomaltulose | 145.8 | 194.4 | 167.4 | 154.8 | 214.2 |
| I-18 | Isomaltulose | 97.2 | 66.6 | 113.4 | 183.6 | 223.2 |
| I-19 | Isomaltulose | 136.8 | 167.4 | 207.0 | 162.0 | 149.4 |
| I-20 | Isomaltulose | 117.0 | 151.2 | 180.0 | 214.2 | 156.6 |
| I-21 | Isomaltulose | 104.4 | 127.8 | 187.2 | 199.8 | 190.8 |
| I-22 | Isomaltulose | 127.8 | 145.8 | 183.6 | 169.2 | 151.2 |
| I-23 | Isomaltulose | 113.4 | 144.0 | 172.8 | 158.4 | 149.4 |
| I-24 | Isomaltulose | 93.6 | 75.6 | 104.4 | 129.6 | 129.6 |
| I-25 | Isomaltulose | 142.2 | 189.0 | 176.4 | 203.4 | 178.2 |
| I-26 | Isomaltulose | 120.6 | 169.2 | 199.8 | 178.2 | 199.8 |
| I-27 | Isomaltulose | 100.8 | 140.4 | 156.6 | 176.4 | 183.6 |
| I-28 | Isomaltulose | 135.0 | 178.2 | 145.8 | 207.0 | 167.4 |
| I-29 | Isomaltulose | 124.2 | 172.8 | 212.4 | 174.6 | 163.8 |
| I-30 | Isomaltulose | 97.2 | 113.4 | 174.6 | 210.6 | 158.4 |
